# Prediction of guidewire-induced aortic deformations during EVAR: a finite element and *in vitro* study

**DOI:** 10.3389/fphys.2023.1098867

**Published:** 2023-07-10

**Authors:** Monica Emendi, Karen H. Støverud, Geir A. Tangen, Håvard Ulsaker, Frode Manstad-H, Pierluigi Di Giovanni, Sigrid K. Dahl, Thomas Langø, Victorien Prot

**Affiliations:** ^1^ Department of Industrial Engineering, University of Rome Tor Vergata, Rome, Italy; ^2^ Department of Health Research, SINTEF Digital, Trondheim, Norway; ^3^ Department of Circulation and Medical Imaging, Norwegian University of Science and Technology, Trondheim, Norway; ^4^ Department of Radiology and Nuclear Medicine, St. Olavs Hospital, Trondheim, Norway; ^5^ HSL S.r.l., Trento, Italy; ^6^ Department of Structural Engineering, Norwegian University of Science and Technology, Trondheim, Norway

**Keywords:** EVAR, finite element, additive manufactured models, intra-operative deformations, abdominal aortic aneurysm

## Abstract

**Introduction and aims:** During an Endovascular Aneurysm Repair (EVAR) procedure a stiff guidewire is inserted from the iliac arteries. This induces significant deformations on the vasculature, thus, affecting the pre-operative planning, and the accuracy of image fusion. The aim of the present work is to predict the guidewire induced deformations using a finite element approach validated through experiments with patient-specific additive manufactured models. The numerical approach herein developed could improve the pre-operative planning and the intra-operative navigation.

**Material and methods:** The physical models used for the experiments in the hybrid operating room, were manufactured from the segmentations of pre-operative Computed Tomography (CT) angiographies. The finite element analyses (FEA) were performed with LS-DYNA Explicit. The material properties used in finite element analyses were obtained by uniaxial tensile tests. The experimental deformed configurations of the aorta were compared to those obtained from FEA. Three models, obtained from Computed Tomography acquisitions, were investigated in the present work: A) without intraluminal thrombus (ILT), B) with ILT, C) with ILT and calcifications.

**Results and discussion:** A good agreement was found between the experimental and the computational studies. The average error between the final *in vitro* vs. *in silico* aortic configurations, i.e., when the guidewire is fully inserted, are equal to 1.17, 1.22 and 1.40 mm, respectively, for Models A, B and C. The increasing trend in values of deformations from Model A to Model C was noticed both experimentally and numerically. The presented validated computational approach in combination with a tracking technology of the endovascular devices may be used to obtain the intra-operative configuration of the vessels and devices prior to the procedure, thus limiting the radiation exposure and the contrast agent dose.

## 1 Introduction

Endovascular aneurysm repair (EVAR) procedure involves the deployment of a stent-graft in the abdominal aorta to exclude the aneurysm from the blood flow. The EVAR procedure consists of several steps that allow the stent graft deployment: first a soft guidewire is inserted in the femoral artery through an access in the groins, then a catheter is pushed over the former guidewire to serve as a guiding rail for the stiff guidewire that straighten the vessel to support the insertion of the stent-graft delivery system (England and Mc Williams, 2013). Traditional methods for instruments guidance during EVAR procedures are based on 2D fluoroscopy and digital subtraction angiography (DSA). These methods expose both the patient and medical staff to X rays and injection of contrast-agent is required for the visualization of the vessels (Rafii-Tari et al., 2013; Maurel et al., 2014b). However, the use of contrast agents can harm the kidneys, thus being dangerous in patients that already present compromised kidneys functions (Faggioni and Mehran, 2016). Moreover, the lack of 3D spatial information can increase the procedural time and make the visualization of intra-operative scenario challenging for the surgeon, especially in case of complex procedures such as juxta-renal and thoracoabdominal aortic aneurysm, where fenestrated and branched stent grafts are needed (Jäckle et al., 2020).

To overcome the challenges with the current EVAR procedures, innovative navigation technologies have been explored, moving towards real-time 3D guidance of the medical instruments (e.g., catheters and guidewires), similar to the ones introduced in other surgical fields (Jäckle et al., 2020). An example is represented by the electromagnetic (EM) tracking technology (de Lambert et al., 2012; Klaassen et al., 2022). Moreover, the integration of pre- and intra-operative acquisitions through image fusion techniques and the introduction of cone beam CT (3D CBCT) in hybrid operating rooms have been shown to improve the surgeon visibility adding the depth perception (Hertault et al., 2014), thus reducing the total amount of contrast agent needed. An accurate registration between intra-operative images and pre-operative 3D models of the patient anatomy is needed (Condino et al., 2014).

To choose the optimal graft model and size, angulation and length of the arteries are measured pre-operatively. As pointed out by recent studies (Gindre et al., 2016; Koutouzi et al., 2019), the insertion of stiff guidewires and endografts delivery systems induces significant deformation of the vasculature. This causes changes in angulations and lengths, thus affecting the accuracy of the pre-operative planning, e.g., stent graft sizing and planned landing zones for the graft (Cercenelli et al., 2018). In addition, the intra-operative deformations make pre-operative images less suitable as a reference for the intra-operative vessel configuration for navigation purposes (Dupont et al., 2021).

Maurel et al. (2014a) measured the *in vivo* deformations caused by the insertion of the stiff guidewire and stent graft delivery system and found that the ostium displacement between the CT angiography (CTA) and contrast enhanced cone beam CT (ceCBCT) images was mostly postero-superior and equal to 6.7 mm (range 2.2–13.5) for the superior mesenteric artery (SMA); 6.2 mm (2.5–13.5) and 6.4 mm (1.9–14.5) for the right and the left renal arteries, respectively; and 5.5 mm (2.3–11.4) for the aortic segment. While Koutouzi et al. (2019), from their comparative study, found a lower tortuosity of the common iliac artery along with a shortening effect in the intra-operative images compared to the pre-operative ones. In detail, they have reported an intra-operative displacement mostly in a cranial direction for the aortic bifurcation and in a ventral direction for the iliac one. Lalys et al. (2019) evaluated how these deformations affects the fusion accuracy and they estimated a mean displacement error of 4.1 ± 2.4 mm at the level of the renal arteries. Moreover, they reported a correlation between the aortic neck angle and the fusion accuracy.

The entity of aortic deformations depends on the patient-specific geometries, on the mechanical properties of the vessels and of the instruments that are inserted. Cercenelli et al. (2018) found in their retrospective study on fluoroscopic images that the effect of straightening the common iliac artery due to guidewire insertion is higher in case of greater tortuosity of the vessels, the highest level of iliac deformations has been observed for cases that present severe tortuosity associated with mild calcifications. Moreover, a higher degree of aortoiliac tortuosity is associated with an increased complexity of endovascular aneurysm repair and a greater risk for the development of iliac injuries (Wolf et al., 2001), preventing in some cases to complete the insertion of the stiff guidewire (Gokani et al., 2012).

To predict and quantify the deformations induced by the insertion of medical instruments inside the vessels, previous works (Kaladji et al., 2013; Gindre et al., 2016; Mohammadi et al., 2018) have developed finite-element (FE) models to simulate the interaction between aorto-iliac structure and guidewires. They have demonstrated the feasibility of the approach to predict the aortic wall and devices deformations during EVAR, with an average error of 2 mm in terms of guidewire path with respect to fluoroscopic images. A detailed computational study on the impact of calcifications on image fusion accuracy, conducted by (McLennan et al., 2021), found a positive correlation between the presence of calcifications and the image fusion accuracy, thus suggesting the importance of including calcifications in numerical models.

Other approaches and technologies have also been considered to tackle the problem: Toth et al. (2015) proposed a skeleton-based ARAP (as-rigid-as-possible) 3D surface mesh deformation approach, that can adapt the preoperative mesh model to the device (reconstructed from the fluoroscopic views), thus, aiming at improving the fusion between pre- and intra-operative images. Breininger et al. (2018) improved the above-described approach, minimizing the user input to one mouse-click (i.e., landmark selection) and considering uncertainties in control points in the deformation correction process. The FE studies compared to the above-mentioned algorithms can predict the deformations due to the procedure and account for the biomechanical aspects such as interactions between vessels and devices (Mohammadi et al., 2018). Therefore, FE simulations could be valuable in the pre-operative planning. However, the current limitation of the FE approach remains the relatively high computational time.

In this study, we used FE and *in vitro* models to analyse the insertion of a stiff guidewire in three cases, modelling the same patient with increasing anatomical complexity. The aim of the present work is to establish a validated simulation tool in a controlled environment, as a starting point for more realistic simulations. To this end, our numerical models were validated against *in vitro* studies. The predictions of the guidewire path and aortic deformations have the potentiality to improve both the pre-operative planning and the intra-operative navigation, and therefore to limit contrast and radiation doses.

## 2 Material and methods

To simulate EVAR and validate it experimentally, the following procedure was considered. First, the patient-specific aortic geometries were segmented from the CTA images to obtain the experimental models through an ad-hoc molding process. The EVAR experiments consisted in acquiring CBCT images of the models before and after the insertion of a stiff guidewire. Second, the materials used to manufacture the models were mechanically tested uniaxially to obtain the material properties used in the FE models. Meshes, boundary and loading conditions were established for the FEA. Lastly, the experimental and FEA configurations were compared. The same procedure was repeated for three different models, based on the pre-operative CTA acquisition of one patient.

Model A: without the intraluminal thrombus (ILT)
Model B: with ILT
Model C: with ILT and calcifications

The details can be found in the following paragraphs.

### 2.1 Image segmentation

The CT angiography of the patient (pixel spacing: 0.5 mm × 0.5 mm; slice thickness: 1 mm) was acquired at St. Olavs Hospital, using a Siemens scanner (Sectra Somatom, Syngo CT) in accordance with a study protocol approved by the regional ethics committee (REK 2016/533, Faculty of medicine, Trondheim, Norway). The patient is a male, 75 years of age at the time of surgery. He had a juxtarenal aortic aneurysm, with a proximal neck length of 9 mm and maximum diameter of 61 mm in reconstructed planes. The patient was operated with a custom-made fenestrated aorto-iliac stent graft from Cook Medical with 3 fenestrations for the right renal artery, left renal artery and superior mesenteric artery, and a scallop for the celiac trunk. The procedure was technically successful, no reinterventions were needed. Written informed consent was submitted by the patient before using the image data to construct the model. A segmentation algorithm, based on intensity threshold and morphological operations, following the work by Lareyre et al. (2019), was written in Python to automatically segment the lumen of the abdominal aorta and the calcifications. For the segmentation of the calcifications, morphological dilate and erode operators were applied to the lumen segmentation, the obtained masks were subtracted to each other with a XOR operator to obtain the region of interest wherein the calcifications were segmented by a threshold greater than 600 HU. Due to the poor contrast with respect to the surrounding tissue the thrombus was segmented manually using the software ImFusion (ImFusion GmbH, Munich, Germany). The obtained segmented 3D geometry was used to manufacture the physical models. The considered geometry has the following characteristic measures: the tortuosity of the iliac arteries, measured following (Piccinelli et al., 2009), are equal to 0.14 and 0.05, respectively, for the left and right iliac artery; the neck angle, defined as in (Kyriakou et al., 2019), is equal to 158°; the volume of calcifications is equal to 1.14 cm^3^. Thus, the relative calcification presence is equal to 3.2%, classified as moderate (McLennan et al., 2021); the ILT percentage volume is equal to 21.4% (Crawford et al., 2016).

For the FEA, the geometries were segmented from the baseline CBCT images of the experiments, using an ad-hoc Python script, based on tresholding and morphological operatotors, i.e., binary opening and closing. The CBCT images were chosen to avoid experimental vs. computational discrepancies on the inital geometry, that can be related to the positioning of the model in the experimental setup and gravitational effects. The geometries of the models can be provided upon request to the corresponding author.

### 2.2 EVAR experiments

#### 2.2.1 Additive manufactured models

**FIGURE 1:**
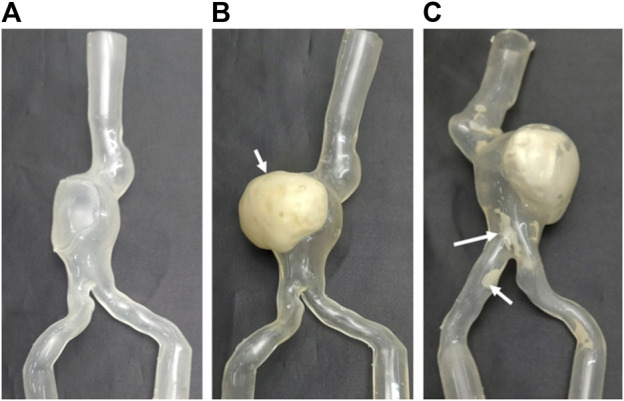
The three manufactured models used for experiments, from left to right **(A)** without intraluminal thrombus (ILT) (Model A), **(B)** with ILT (indicated by the white arrow) (Model B), **(C)** with ILT and calcifications (indicated by white arrows) (Model C).

The models were manufactured following an ad-hoc molding procedure: the inner mold was 3D printed using stereolitography (SLA) technology, the outer cast was obtained in silicone. The chosen material for the aortic wall, a transparent polyurethane resin (Synthene, shore hardness 30), obtained by mixing two different components, was poured between the inner and outer casts under vacuum and cured at a temperature of 70°. Then, the inner cast was manually removed to obtain Model A, with a thickness of 2.5 mm. Model B, with the addition of ILT, was obtained as follows: the thrombus was firstly 3D printed with SLA resin, used as master for making the silicone mould, a 3-component soft polyurethane (UPX 8400-1, 25 shore A hardness) was then vacuum casted. The ILT model was glued onto the inner aortic mould (3D printed as for Model A). Synthene was poured in the assembled mould, and then left to cure at 70° for 2 h. Model C, with the inclusion of calcifications, was realized following the same procedure described above. In addition, for Model C, the calcifications were 3D printed with SLA (material: Somos ProtoGen 18420 photopolymer) and anchored to the inner mould through pins to remain embedded in the aortic wall after the casting procedure. In Figure 1, the three different models are shown.

#### 2.2.2 Experimental setup

**FIGURE 2:**
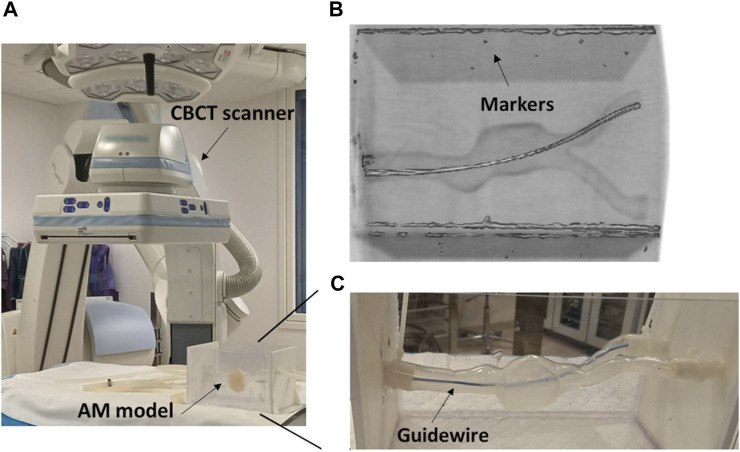
**(A)** Detail of the CBCT scanner in the hybrid operating room, with the model placed in a rigid box. **(B)** Example of a CBCT acquisition, with the reference markers used for registration. **(C)** Detail of the additive manufactured (AM) model (Model A) with the guidewire fully inserted.

The experiments were carried out in the hybrid operating room at St. Olavs Hospital, Trondheim, Norway. The images of the models were acquired with a C-arm CBCT scanner (Artis Zeego, Siemens, Erlangen, Germany), spatial resolution = 0.7 mm × 0.7 mm (Figure 2A). For each model a CBCT was acquired at baseline conditions and a new acquisition after inserting a stiff guidewire (Back-up Meier, Boston Scientific), from the left iliac artery. This is one of the stiffest guidewires used during EVAR (Harrison et al., 2011) and it has a floppy tip to ease the navigation and prevent damages on the vessel wall. The models were placed in a rigid box attached through their extremities, as shown in Figure 2C. The inner surfaces were wet with slippery fluid (a solution of water and glycerol) to facilitate the navigation. For each model the acquired CBCTs were registered with respect to the baseline acquisition. A rigid landmark registration was performed with CustusX software (Askeland et al., 2016) using the tantalum spherical markers glued to the rigid box (Figure 2B). The baseline and the deformed configurations were then segmented using an in-house Python script. To obtain the *in vitro* deformations, the difference between the baseline and final configurations (*d*_EXP_) was quantified using the Hausdorff distance tool available in the software Meshlab (Cignoni et al., 1998). *d*_EXP_ is the shortest distance of a point on the deformed segmented surface to the undeformed (baseline) segmented surface.

Moreover, the deformations of the iliac arteries were measured as change in their tortuosity. This was done with the VMTK extension in 3D Slicer (Kikinis et al., 2014), that implements the following definition (Piccinelli et al., 2009):

$$\chi =\frac{L}{D}-1,$$ (1)

where L is the length of the centerline and D the Euclidean distance between its endpoints. Thus, the percentage of iliac arteries deformations, similarly to (Cercenelli et al., 2018), is obtained by:

$$\delta \%=\frac{\chi_{\text{def}}-\chi_{\text{und}}}{\chi_{\text{und}}}\%,$$ (2)

where *χ*_def_ is the tortuosity of the deformed configuration, with the guidewire fully inserted, and *χ*_und_ is the tortuosity of the undeformed configuration.

### 2.3 Mechanical testing

**FIGURE 3:**
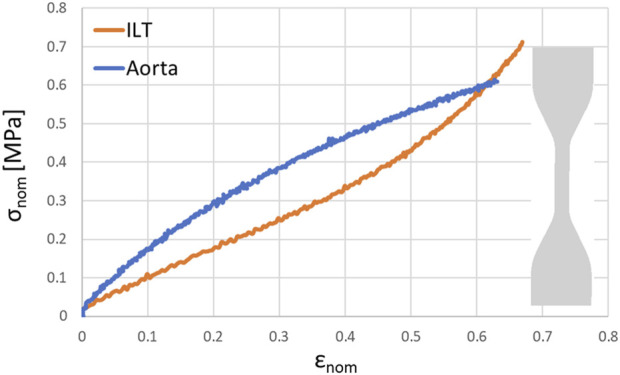
Resulting stress (*σ*_nom_)—strain (*ϵ*_nom_) curve obtained from uniaxial loading test of the materials used to manufacture the aorta and the ILT (with a detail of the specimen used for uniaxial loading test).

To assess the mechanical properties of the materials used to manufacture the aortic wall and ILT, a set of uniaxial traction tests were performed on three dog-bone specimens for each material following the ASTM D412 standard. The specimens were obtained from a silicone molding procedure. The details of the testing procedure are described in Gasparotti et al. (2019). The resulting average strain-stress curves of the aortic and ILT materials are plotted in Figure 3. Since their mechanical behaviour, at least for small strains, was well approximated by a linear function, a linear elastic material model was chosen for the FEA.

### 2.4 Finite element simulation

We herein performed an explicit FEA of the insertion procedure of the stiff guidewire using the commercial FE software LS-DYNA (Ansys, Canonsburg, Pennsylvania, United States). The details are described in the following paragraphs.

#### 2.4.1 Discretization

**FIGURE 4:**
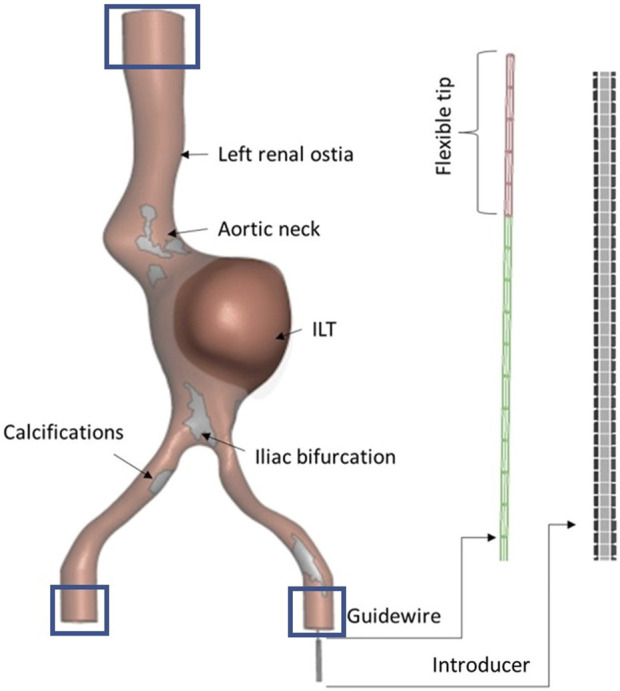
Details of the parts of the computational model: aorta with anatomical details, guidewire with flexible tip and introducer. The displacement of the nodes enclosed in the rectangular area were constrained (Boundary conditions).

The geometries were segmented from the experimental CBCT baseline acquisitions, as described in Section 2.1. The aortic wall was meshed with triangular shell elements with 2.5 mm thickness (C^0^ LS-DYNA type). The formulation of the C^0^ triangular shell is due to Kennedy, Belytschko, and Lin (Kennedy et al., 1986). The element characteristic length of 1.4 mm for the aorta was chosen upon a mesh sensitivity analysis study performed on model A, described in Section 3.2. For the Back-Up Meier guidewire, beam elements (Hughes-Liu with cross section integration), with a characteristic length of 4 mm, were chosen. The Hughes-Liu beam element is based on a degeneration of the isoparametric 8-node solid element, as suggested by Ahmad et al. (1970). A rigid introducer, discretized with shell elements, was included to avoid undesired movements of the guidewire outside the vessel. For model B and C, the ILT was discretized with tetrahedral elements with a characteristic dimension of 1.2 mm. In addition, for model C, the calcifications were modeled with shell elements of 1.2 mm length, similarly to previous studies (Dupont et al., 2021; McLennan et al., 2021). The simulated components are shown in Figure 4. The information on the element type for the different parts are summarized in Table 1.

**TABLE 1:** Details of the FEA setup.

	Aorta	Guidewire	Introducer	ILT	Calcifications
Element type	Shell	Beam	Shell	Tetrahedra	Shell
Element size [mm]	1.4	4	1	1.2	1.2
Young’s modulus [MPa]	1.5	1.4e5	1e5	0.8	2250
Poisson’s ratio	0.4	0.3	0.3	0.4	0.4

#### 2.4.2 Material properties

The aortic wall was modeled as a linear elastic material, with a Poisson’s ratio of 0.4 and a Young’s Modulus of 1.5 MPa, based on the uniaxial mechanical test described in Section 2.3. The Back-Up Meier guidewire was modeled as a linear elastic material, with Young’s Modulus E = 140 GPa (Harrison et al., 2011) and Poisson’s ratio equals to 0.3. The floppy tip of the guidewire was modeled with a gradually decreasing elastic modulus, ranging from 1 to 50 GPa, (Gindre et al., 2015). The calcifications were modeled as linear elastic, with a Young’s modulus of 2,250 MPa and a Poisson’s ratio of 0.4, obtained from the data sheet of the material used to manufacture them. For the ILT, a Poisson’s ratio of 0.4 and a Young’s modulus equals to 0.8 MPa, obtained from the uniaxial mechanical characterization, was assigned. The material properties for the different parts are summarized in Table 1.

#### 2.4.3 Numerical set-up

For dynamic explicit analyses in LS-DYNA, the displacements are updated using the central difference time integration scheme. For further details, we refer to the LS-DYNA Theory manual (Hallquist, 2006).The reason for using an explicit solver in this work is its effectiveness in handling the nonlinearities due to the large displacements and the contact conditions between the different parts of our models.

To limit the oscillations, dampers were added at each node of the aorta, with a damping coefficient of 10^–7^. To decrease the computational time, a mass-scaling method consisting in artificially increasing the guidewire density was applied, similarly to (Gindre et al., 2015). Thus, the initial time step was equal to 5.10^–6^ s. The simulations’ total time, i.e., the duration of the insertion, was set to 1.2 s and it took about 40 min to run the entire simulation on 2 CPUs for model A (Intel Xeon Gold 6152 CPU @ 2.10 GHz).

#### 2.4.4 Loading, boundary conditions and contact

The insertion of a stiff guidewire in the left iliac artery was simulated imposing a velocity *v*(*t*) to the most distal node located at the bottom of the guidewire, as suggested by (Gindre et al., 2015):

$$\begin{array}{c} v\left(t\right)=V_{0}t^{3}\left(10-15t+6t^{2}\right),t\in \left[T1,T2\right]\\ v\left(t\right)=V_{0},t\in \left[T2,T3\right]\\ v\left(t\right)=V_{0}-V_{0}t^{3}\left(10-15t+6t^{2}\right),t\in \left[T3,T4\right] \end{array}$$ (3)

where *V*_0_ = 500 mm/s and T1, T2, T3, T4 are subdivisions of the total time of the simulation, and equal to 0, 0.4, 0.8 and 1.2 s, respectively. The velocity vector had the same direction as the guidewire.

Note that a sensitivity analysis was performed to ensure that the velocity values were not affecting the guidewire equilibrium position and the aortic deformations.

The translational degrees of freedom of the nodes of the proximal and distal endings of the vessel were constrained to reproduce the experimental conditions, as shown by the boxes in Figure 4.

Following previous works (Gindre et al., 2015; Mohammadi et al., 2018), a frictionless contact algorithm (Automatic beams to surface LS-DYNA type), based on soft constraint penalty formulation, where the interface stiffness is based on the nodal mass and the global time step size (Hallquist, 2006), was applied between the guidewire and the vessel. While a standard penalty formulation contact type was considered between the guidewire and the introducer. For Models B and C, LS-DYNA tied shell edge to solid contact option was selected to anchor the ILT to the lumen. For model C, a node-merging operation was performed to tie the calcifications to the aortic wall.

Moreover, the effect of varying the initial position of the guidewire on the final aortic configuration was investigated, along with the effect of varying the stiffness of the guidewire and the friction coefficient between the guidewire and the aortic wall, as shown in Section 3.2.

### 2.5 *In vitro* vs. *in silico* comparison

The difference between the initial and the final aortic configuration obtained from FEA (*d*_FEA_), also referred to as deformations, was calculated as described in 2.2.2 for *d*_EXP_. Therefore, *d*_FEA_ is the shortest distance of a point on the deformed FE final configuration to the undeformed FE configuration. Note that *d*_FEA_ does not represent the displacements of the FE nodes. It is used instead for comparison with the experimental results.

The deformations of the iliac arteries were calculated as variation of their tortuosity, as described in 2.2.2. The resulting FE deformations were validated against the experimental ones (for Models A, B and C). The considered metrics of comparison between the final aortic deformations were: the distance between the experimental and numerical final configurations, also referred to as error, *e*_aorta_, computed as described in 2.2.2; the DICE similarity coefficient and the relative overlap (Taha and Hanbury, 2015). These last two metrics were obtained as follows: the CBCT segmentations were converted to 3D labels, that were used to calculate the metrics with the software ImFusion (ImFusion GmbH, Munich, Germany).

In addition, the experimental path of the guidewire, segmented by tresholding from the CBCT acquisition, was compared to the final path of the guidewire obtained from FEA, in terms of Hausdorff distance (Cignoni et al., 1998), *e*_guidewire_, similarly to the approach followed for the aortic wall.

## 3 Results

### 3.1 Experimental results

From the experiments with the guidewire inserted in the left iliac artery, the deformations *d*_EXP_ from the initial to the final aortic configurations are respectively equal to:

Model A: *d*_EXPmax_ = 5.27 *mm*, *d*_EXPmean_ = 1.41 *mm*,
Model B: *d*_EXPmax_ = 9.08 *mm*, *d*_EXPmean_ = 1.60 *mm*,
Model C: *d*_EXPmax_ = 12.80 *mm*, *d*_EXPmean_ = 2.71 *mm*.

For all the models, the maximum values of *d*_EXP_ are located anteriorly at the level of the upper left iliac artery towards the iliac bifurcation. Experimentally the inclusion of the thrombus leads to an increase in maximum deformations of 42% compared to Model A, while the inclusion of thrombus and calcifications leads to an increase in max deformations of 59% compared to Model A.

**FIGURE 5:**
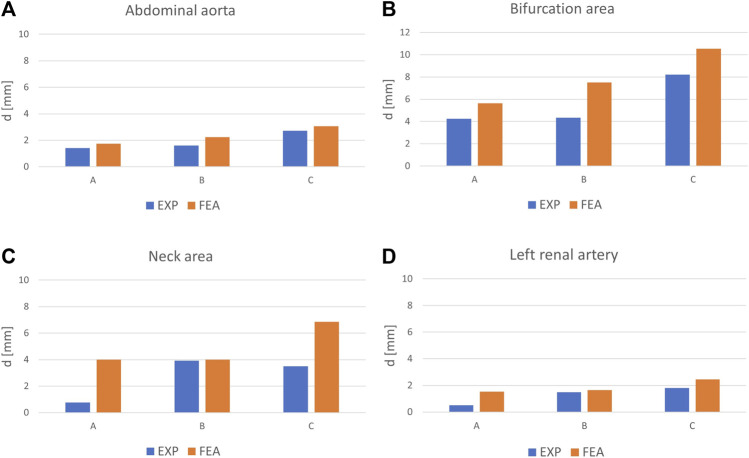
Average values of deformations, d from experiments and FEA for the entire abdominal aorta **(A)** and for different anatomical positions: bifurcation region **(B)**, aortic neck **(C)** and ostium of the left renal artery **(D)**; for Model A without ILT, Model B with ILT, Model C with ILT and calcifications, indicated as A, B, C in the graphs.

**FIGURE 6:**
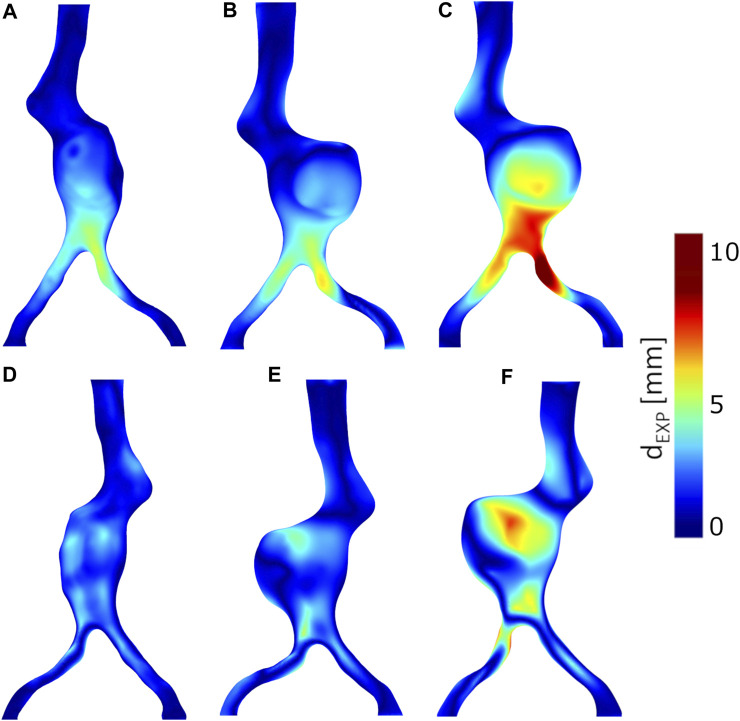
Experimental deformations from CBCTs, *d*_EXP_. Anterior views: **(A)** Model A without thrombus, **(B)** Model B with thrombus, **(C)** Model C with thrombus and calcifications. Posterior views: **(D)** Model A without thrombus, **(E)** Model B with thrombus, **(F)** Model C with thrombus and calcifications.

The displacements of the anatomical points of interest: iliac bifurcaton, aortic neck (inner curvature), renal ostia are displayed in Figure 5. The complete color maps in terms of *d*_EXP_ are reported in Figure 6.The left iliac artery has an initial value of tortuosity of 0.14, after the insertion of the stiff guidewire it straightens, reaching values of tortuosity *χ* of 0.050, 0.056 and 0.047, respectively, for Models A, B and C (with a percentage change *δ%* of 64.3%, 59.9%, 65.9%). For the right iliac artery, the initial value of tortuosity equals to 0.05 becomes 0.030, 0.025 and 0.034 for Models A, B and C, respectively, with a percentage change *δ%* of 40%, 48.9% and 31.2%.

### 3.2 Finite element analysis results and sensitivity analysis

From the simulation of guidewire insertion in the left iliac artery, we obtain that the maximum and average deformations (*d*_FEA_), computed between the initial and the final aortic configuration, are equal to:

Model A: *d*_FEAmax_ = 7.45 *mm*, *d*_FEAmean_ = 1.75 *mm*,
Model B: *d*_FEAmax_ = 10.41 *mm*, *d*_FEAmean_ = 2.24 *mm*,
Model C: *d*_FEAmax_ = 12.70 *mm*, *d*_FEAmean_ = 3.06 *mm*.

For all the models, the maximum values of *d*_FEA_ are located anteriorly at the level of the upper left iliac artery towards the iliac bifurcation, as in the experimental results.

The inclusion of the thrombus leads to an increase in maximum deformations of 28% compared to Model A, while the inclusion of thrombus and calcifications leads to an increase in max deformations of 41% compared to Model A.

**FIGURE 7:**
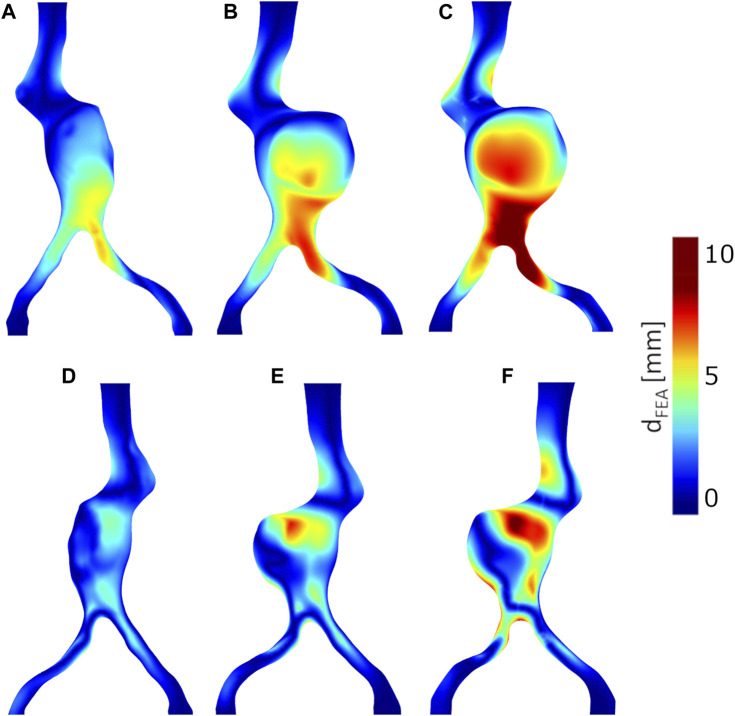
Deformations obtained from FEA, *d*_FEA_. Anterior views: **(A)** Model A without thrombus, **(B)** Model B with thrombus, **(C)** Model C with thrombus and calcifications. Posterior views: **(D)** Model A without thrombus, **(E)** Model B with thrombus, **(F)** Model C with thrombus and calcifications.

The displacements of the anatomical points of interest obtained from the FEA: iliac bifurcaton, aortic neck, renal ostia are plotted in Figure 5. The complete color maps in terms of *d*_FEA_ are reported in Figure 7.

**FIGURE 8:**
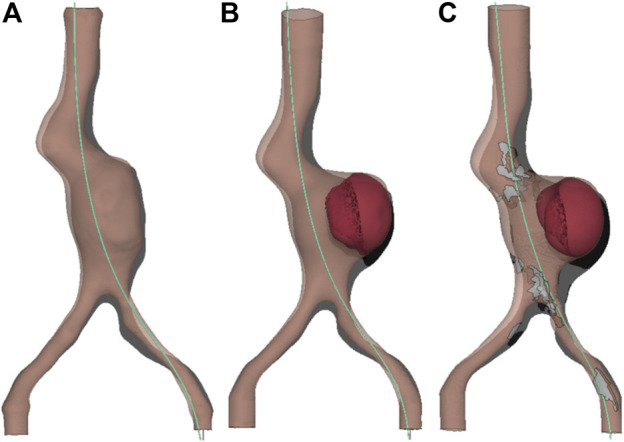
FEA Results: path of the guidewire (light blue), initial aortic configuration (grey), final deformed aortic configuration (pink). Anterior view, frontal plane. **(A)** Model A without ILT, **(B)** Model B with ILT, **(C)** Model C with ILT and calcifications.

The left iliac artery, with an initial value of tortuosity of 0.14, is straightened by the insertion of the stiff guidewire, reaching values of tortuosity *χ* of 0.03, 0.046 and 0.042, respectively, for Models A, B and C (with a percentage change *δ%* of 78.6%, 66.8% and 70.2%). For the right iliac artery, the initial value of tortuosity equal to 0.05 becomes 0.03, 0.027 and 0.04 for Models A, B and C, respectively (with a percentage change *δ%* of 40%, 46.7% and 20.5%). The initial aortic configurations, the deformed ones and the paths of the guidewire are reported in Figure 8 for the three considered models.

The sensitivity analysis on the aortic shell size was carried out using the maximum displacement as a metric. The chosen mesh size, i.e., 1.4 mm, is a trade-off between the accuracy and the computational cost. The error on the maximum displacement compared to the finest mesh (0.7 mm) is equal to 2.3% and the computational time is about 20% of the time needed to compute the simulation with the finest mesh. The detailed results are reported in Table 2.

**TABLE 2:** Sensitivity analysis on the aortic wall mesh.

Average mesh size [mm]	Error max displacement [%]	Computational time
2.8	14.8	17 min 7 s
1.4	2.3	41 min 24 s
0.7(iliacs)/1.4	2	1 h 34 min 3 s
0.7	—	3 h 32 min 20 s

**FIGURE 9:**
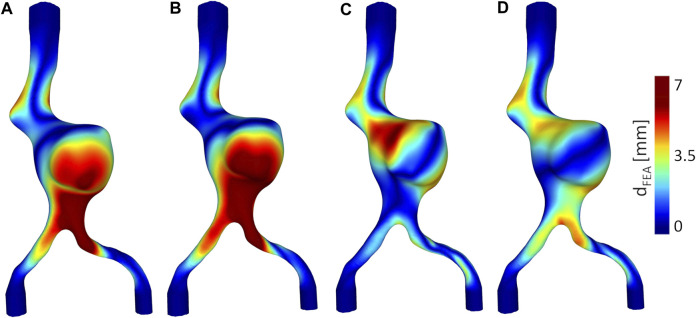
From left to right, deformations *d*_FEA_ between the initial vs. final configuration when the initial position of the guidewire is **(A)** centered on the iliac inlet, in a vertical position (0°inclination in frontal and sagittal planes) **(B)** 20°counterclockwise inclination in frontal plane, **(C)** 25°clockwise inclination in sagittal plane, **(D)** 20°clockwise inclination in frontal plane.

A sensitivity analysis of the insertion position and angulation of the guidewire was carried out. For model A, with a rotation angle of 30°, in the sagittal plane, the maximum difference in terms of aortic deformations is equal to 6.1 mm, while with a rotation of 30° in the frontal plane differences are equal to 2.5 mm at maximum. For Model B, the results are reported in Figure 9.

Considering a friction coefficient of 0.2 (He et al., 2020) compared to a frictionless contact results in a difference on the aortic maximum deformations *d*_FEAmax_ equal to a 0.5 mm (i.e. 10% error) for model A. For completeness, we report here also the comparison between a frictionless contact and a friction coefficient of 0.5, the maximum error obtained in this case is equal to 1.03 mm, while the average is equal to 0.35 mm.

For Model A, the results with three different values of stiffness of the guidewire: 60 GPa, 140 GPa and 158 GPa, inserted from the left iliac artery, were compared. These values correspond to three different guidewire types that can be used during the procedure: the Amplatz (Boston Scientific), the Back-Up Meier (Boston Scientific) and the Lunderquist (Cook Medical), respectively, (Harrison et al., 2011). Comparing the Amplatz to the Lunderquist guidewire, the maximum difference, located at the posterior side of the left iliac artery near the bifurcation region, is equal to 5.16 mm. The maximum difference between the final aortic configurations with the Amplatz vs. the Back-Up Meier guidewires inserted is equal to 4.36 mm, located in the same area as for the previous comparison. For the comparison Lunderquist vs. Back-Up Meier the maximum difference is equal to 0.82 mm, located in the same region as in the previous analysis.

### 3.3 *In vitro* vs. *in silico* comparison

**FIGURE 10:**
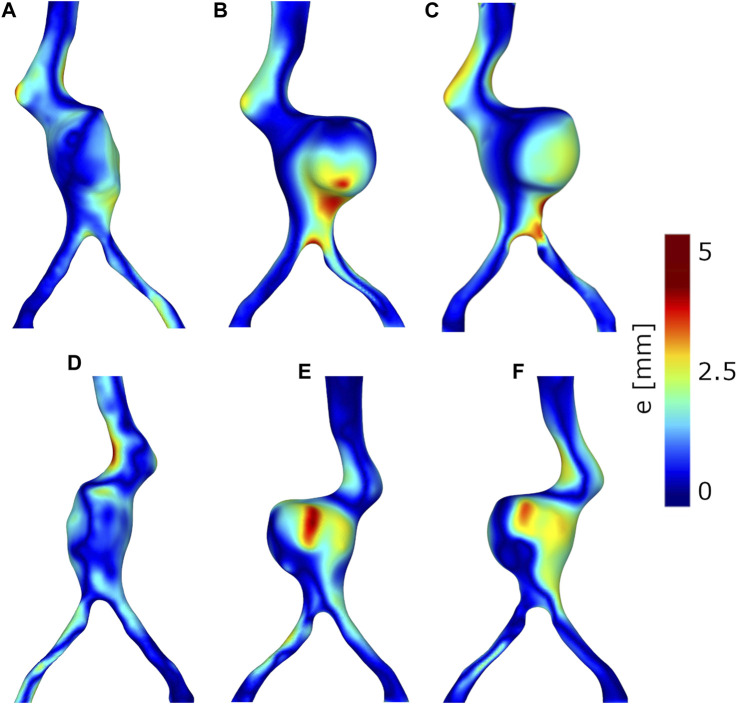
Error (*e*_aorta_) between the simulated and experimental final aortic configurations. Anterior views: **(A)** Model A without thrombus, **(B)** Model B with thrombus, **(C)** Model C with thrombus and calcifications. Posterior views: **(D)** Model A without thrombus, **(E)** Model B with thrombus, **(F)** Model C with thrombus and calcifications.

The main results in terms of aortic deformations and change in tortuosity, obtained from the experiments and the FEA are compared in Table 3. The average errors (*e*_aorta_) between the final experimental and FEA aortic configurations for Models A, B and C are equal to 1.17 mm, 1.22 mm and 1.40 mm, respectively. The maximum errors are respectively equal to 4.76, 5.14 and 5.18 mm. The map of the errors (*e*_aorta_) is plotted in Figure 10.

**TABLE 3:** Maximum and average values of experimental and numerical guidewire-induced deformations, **d**_**EXP**_ and **d**_**FEA**_, respectively. Percentage change in tortuosity for left and right common iliac arteries, calculated from the experiments and from FEA, ***δ***_**EXP**_ and ***δ***_**FEA**_.

	d_EXP_[mm]		d_FEA_[mm]		*δ*_EXP_[%]		*δ*_FEA_[%]	
Model	max	mean	max	mean	left iliac	right iliac	left iliac	right iliac
A	5.27	1.41	7.45	1.75	64.3	40.0	78.6	40.0
B	9.08	1.60	10.41	2.24	59.9	48.9	66.8	46.7
C	12.80	2.71	12.70	3.06	65.9	31.2	70.2	20.5

The considered models were divided in 4 regions: 1) neck region 2) aneurysmatic region 3) left iliac and 4) right iliac. For each region the average diameter was calculated using the VMTK extension within 3D Slicer (Kikinis et al., 2014). The relative error for each region was calculated as the percentage ratio of the average error over the average diameter. The same procedure was repeated for model A, B, C. The relative errors are.

For model A), 1) 7,88% 2) 4,44% 3) 15.43% 4) 5.28%;
For model B), 1) 6.84% 2) 4.97% 3) 8.87% 4) 10.77%;
For model C), 1) 8.85% 2) 5.93% 3) 9.69% 4) 6.21%.

For Models A, B and C the Dice similarity coefficient between the final aortic configurations are, respectively, equal to 88.9%, 90.31%, 89.1%. The values of relative overlap are respectively equal to 89.8%, 91.45%, 89,09%.

Moreover, we compared the final experimental and numerical paths of the guidewire in terms of *e*_guidewire_. For Model A, the average value of *e*_guidewire_ is equal to 2.48 mm, while the maximum is equal to 6.9 mm, with larger discrepancies in the antero-posterior direction starting from the aortic neck. For Model B, the average *e*_guidewire_ is equal to 2.52 mm and the maximum is equal to 6.66 mm. Larger discrepancies are located towards the upper part of the aorta starting from the aortic neck, in the antero-posterior direction, similarly to Model A. For Model C, the average value of *e*_guidewire_ is equal to 1.48 mm and the maximum is equal to 3.93 mm. The greatest discrepancy is in the left-right direction, in the aneurysmatic region.

## 4 Discussion

The results of the simulation and the experiments agree that an introduction of the guidewire from the left side causes a displacement of the aorta towards the upper right direction. The left side was chosen to insert the guidewire for two main reasons: it is the most common reported access site for stent graft delivery in EVAR (Cercenelli et al., 2018) and it is the most tortuous path for the studied geometry. The effect of straightening of the iliac arteries is captured from both the experimental and computational studies, with similar values of variations in tortuosity. As expected for this case study, the left iliac artery being the more tortuous and coinciding with the side of insertion, presents the largest decrease in tortuosity. The *in vitro* and *in silico* results show similar patterns of deformations, with higher values located at the anterior side of the iliac artery in which the guidewire was inserted, towards the bifurcation region. The two approaches also agree that the deformation values increase going from Model A to Model C, with the addition of ILT and calcifications, suggesting the importance of their inclusion in this type of analysis. The deformations obtained from FEA are in average greater than the experimental ones for all the models, as it can be seen from Figure 6, Figure 7, and around the bifurcation, the neck and the renal artery as shown in Figure 5. The discrepancies between the two approaches may be due to segmentation and registration inaccuracies of the CBCTs (mean accuracy of landmark based registration = 0.52 mm), which are also affected by the limited resolution of CBCTs and metal artefacts in the images caused by the stiff guidewire. In addition, as shown in Figure 9, there are uncertainties related to the insertion angle and position of the guidewire. In future we plan to record this information during the experiments. It has to be noted that some reasonable assumptions were made for the following parameters: the mechanical properties of the guidewire tip and the friction coefficient between the aorta and the guidewire. The mechanical properties of the tip of the considered guidewire, due to the absence of data in literature, were approximated with values from another guidewire type (Gindre et al., 2015; Dupont et al., 2021). However, this assumption is not affecting the results. Indeed, the stiff part of the guidewire is the one that has the impact on the aortic deformations, as shown in our sensitivity analysis study. There are some uncertainties related to the friction coefficient, whose value has not been assessed through experimental test for the considered materials. Nevertheless, we assessed that, at final equilibrium condition, the impact of a friction coefficient up to 0.5 was negligible on the results of interest. Thus, we reasonably assumed a frictionless contact type.

The displacements of the anatomical landmarks are on average smaller than the one reported in literature (Maurel et al., 2014a). Some possible reasons may be: the literature data refer to deformations caused by both the guidewire and the stent graft delivery system, the material considered for the model could be stiffer than the real one, and the geometry less tortuous in average than those considered in the cited study. In agreement with a recent study of (McLennan et al., 2021), we found that the calcifications have a non-negligible impact on the guidewire induced aortic deformations. Additionally, the ILT has an effect on the deformations, although minor, at least for the considered geometry. To the best of our knowledge, there are no previous works that have investigated the effect of ILT presence on intra-operative deformations. Some studies (Mohammadi et al., 2018), (McLennan et al., 2021) considered it but without investigating its effect, i.e., there is no comparison with the corresponding model without ILT.

Limitations of the present work include the simplifications with respect to the *in vivo* scenario: considering the mechanical role of the surrounding tissues (spine and abdominal fat), although adding complexity to the models, could be beneficial to obtain more realistic deformation patterns, as suggested by previous works (Gindre et al., 2015; Mohammadi et al., 2018). In this case, a comparison with intra-operative data will be desirable.

Regarding the material properties of the models, the material chosen for the aorta has a Young’s modulus of 1.5 MPa, in agreement with the study from (Vallabhaneni et al., 2004), that reported that the tissue of abdominal aortic aneurysm (AAA) has a mean Young’s modulus of 1.8 MPa (0.16–4.52 MPa) at stresses experienced *in vivo*. The assumption of linear elasticity is valid and accurate enough to describe the behavior of the additive manufactured model. Nevertheless, we are aware that to truly capture the aortic tissue behavior a hyperelastic anisotropic model is more appropriate, but out of the scope of the present work. In future we aim to assess the impact of this choice on our results. For the ILT, the study of (Wang et al., 2001) reports that it consists of three layers (luminal, medial and abluminal) and the Young’s moduli for the luminal and medial layers are respectively 0.54 MPa and 0.28 MPa. In this study, we chose a single layer model for simplicity with a Young’s modulus of 0.8 MPa, slightly greater than the value for the luminal layer of ILT. The calcifications have a Young’s modulus (2,250 MPa) higher than the one reported in literature (50 MPa) (McLennan et al., 2021). The choices of the material were indeed a compromise between the manufacturing-experimental needs and realism.

The described computational approach has been used in literature (Gindre et al., 2015; Mohammadi et al., 2018). Nevertheless, the aim of the present work is to use it as a starting point for further parametric analyses and comparisons with experiments and alternative methods, e.g., shortest path algorithm for the prediction of the guidewire’s path.

In future we plan to include a larger population study, focusing on complex, tortuous geometries, and to investigate the possibility of running the simulations in real-time.

Reproducing the real *in vivo* behaviour is out of the scope of the present work, being its aim the validation of EVAR numerical simulation in a controlled environment. Previous works focus on validation with respect to intra-operative data and in terms of the guidewire path (Gindre et al., 2015), while this work focuses on validation with respect to *in vitro* data, both in terms of deformations and guidewire path.

## 5 Conclusion

In this preliminary study a validated numerical approach for the prediction of guidewire-induced deformation in models of AAA of different anatomical complexity was achieved. This may be beneficial in the pre-operative planning for the choice of the optimal stent graft and landing zones. In addition, the *in silico* deformation model could be implemented in patient-specific virtual reality EVAR simulators, whose current major limitation is the lack of biomechanical interactions between the vessel and the devices (Cercenelli et al., 2018). The obtained models and the experimental setup could also be used for training purposes of the clinical personnel for EVAR simulations, stent graft deployment, image acquisition with complex systems, e.g., CBCT with rotating C-Arm, and for testing innovative navigation technologies, e.g., EM tracking and shape sensing systems. The presented computational approach used in combination with a tracking technology, can potentially display a continuously updated 3D anatomical map to the operator during EVAR, without the use of X-ray radiation and contrast agent.

## Data Availability

The data analyzed in this study is subject to the following licenses/restrictions: Ethical restrictions. Requests to access these datasets should be directed to ME, monica.emendi@students.uniroma2.eu.
